# Behavior-Oriented Nomogram for the Stratification of Lower-Grade Gliomas to Improve Individualized Treatment

**DOI:** 10.3389/fonc.2020.538133

**Published:** 2020-12-17

**Authors:** Ruo-Lun Wei, Li-Wei Zhang, Jian-Guo Li, Feng-Dong Yang, Ya-Ke Xue, Xin-Ting Wei

**Affiliations:** ^1^ Department of Neurosurgery, The First Affiliated Hospital of Zhengzhou University, Zhengzhou, China; ^2^ Department of Neurosurgery, West China Hospital, Sichuan University, Chengdu, China

**Keywords:** glioma, lower-grade glioma, nomogram, recurrence, O-6-methylguanine-DNA methyltransferase, secondary glioblastoma, temozolomide

## Abstract

Secondary glioblastomas (sGBM) are derived from previously lower-grade [World Health Organization (WHO) grades II or III] gliomas. Lower-grade benign-behaving gliomas may retain their former grade following recurrence, or may become malignant higher-grade glioblastomas. Prediction of tumor behavior in lower-grade gliomas is critical for individualized glioma therapy. A total of 89 patients were included between January 2000 and January 2019 in the present study to establish a nomogram via univariate and multivariate logistic regression analyses. Nomogram predictive performance was tested in the validation group. We then analyzed 36 O-6-methylguanine-DNA methyltransferase (*MGMT*) unmethylated lower-grade gliomas from patients seen at West China Hospital of Sichuan University. Survival statistics were calculated with the Kaplan-Meier method. Two clinical factors (molecular diagnosis and WHO grade), five radiological factors (location, cortical involvement, multicentricity, uniformity, and margin enhancement), one biomarker (*1p19q* codeletion), and a combination of three biomarkers (*IDH*+/*ATRX*-/*TP53*-) were associated with glioma upgrading. Nomograms positive for these prognostic factors had an AUC of 0.880 in the derivation group and 0.857 in the validation group. The calibration and score-stratified survival curves for the derivation group and validation group were good. An operational nomogram was published at https://warrenwrl.shinyapps.io/DynNomapp/. The overall survival of secondary gliomas in the MGMT-unmethylated cohort were influenced independently by the use of temozolomide during the treatment of formerly low-grade gliomas (*p*=0.00096). Clinical and radiological factors and biomarker-based behavior-oriented nomograms may offer a feasible identification tool for the detection of sGBM precursors. This method may further assist neurosurgeons with the stratification of lower-grade glioma cases and thus the development of better, more individualized treatment plans.

## Introduction

Gliomas are among the most common adult brain tumors. Due to their diffusely infiltrative nature and significant molecular and cellular heterogeneity, glioma tumor recurrence is almost inevitable in most cases (1). Unlike other cancers, the histological and staging classification of glioblastomas follows a unique grading system. For considerable number of gliomas, recurrence is inevitable. Lower grade (WHO grades II or III) benign-behaving gliomas been resected in the former surgery may recur in their former grade, or become higher-grade, malignant glioblastomas, as the secondary glioblastoma (sGBM). Because of a lack of adequate overall survival (OS)-oriented studies, the prediction of lower-grade glioma behavior is difficult, thus rendering individualized glioma therapy more difficult.

To this end, in the present study we created and validated a nomogram to distinguish lower-grade gliomas with certain clinical, radiological, and biomarker features. This work also illustrates that temozolomide (TMZ) use in unmethylated *MGMT* lower-grade gliomas may benefit the OS after tumor recurrence. To our knowledge, this is the first behavior-oriented prediction model stratifying lower-grade gliomas and providing clinical utility for individualized treatment.

## Materials and Methods

### Nomogram Cohort

A total of 125 patients between January 2000 and January 2019 were included in the study. In the nomogram cohort, 89 patients were recruited from the First Affiliated Hospital of Zhengzhou University. For the survival analysis cohort, 36 patients were recruited from the West China Hospital of Sichuan University. Study inclusion criteria were as follows: 1) a prior primary surgery for WHO grades II or III glioma and surgery for recurrent lesions, followed by histopathology, 2) gross total resection or subtotal resection during the primary surgery, 3) complete medical records, and 4) a follow-up interval of at least 24 months. Exclusion criteria were the following: 1) tumor specimen insufficient for building a tissue microarray, 2) a partial resection or biopsy performed during the primary surgery, 3) incomplete medical records, and 4) loss to follow-up. The following clinical characteristics were recorded: 1) age, 2) gender, 3) date of diagnosis, 4) surgery performing date, 5) adjuvant therapy, and 6) date of death. Approval for this retrospective, Health Insurance Portability and Accountability Act-compliant study was obtained from the medical ethics committees at both institutions.

Resection degree was determined by comparing post-operative and pre-operative imaging. Gross total resection was defined as leaving no residual tumor. Subtotal resection was defined as leaving less than 5% residual tumor. Tumor volume was estimated using the Coniglobus formula (length × width × height/2).

### Histological Evaluation

Histological evaluations were performed on H&E stained archival slides. All cases were reviewed by neuropathologists according to 2016 WHO classification criteria of tumors of CNS.

### Immunohistochemical Staining and Analyses 

An automated immunohistochemical staining system (BenchMark XT, USA) was used to perform immunohistochemical staining. Stained tissue microarray slides were scanned with a Leica Aperio AT2 scanner at 400× magnification. Digital images were analyzed by Leica Aperio ImageScope software with the Nuclear v9 algorithm. The following biomarkers were recorded: 1) *IDH1 (isocitrate dehydrogenase 1)*, 2) *1p19q*, 3) *TP53*, 4) *ATRX*, and 5) *Ki67*. *IDH1* and *1p19q* codeletion and alpha thalassemia/mental retardation syndrome X-linked (*ATRX)* status were scored as positive or negative. *TP53* status was quantified as the percentage of stained nuclei: less than 10% of stained nuclei indicate an absence of immunoreactivity; 10-30% indicate a score of 1+; 30.1%–50% indicate a score of 2+; and more than 50% indicate a score of 3+. Scores of –1 or 1+ were regarded as *TP53* negative, and 2+ and 3+ were regarded as *TP53* positive. A *Ki67* index was also calculated according to the percentage of *Ki67*-positive tumor cells present in the sample.

### MRI Protocol and Post-Processing

We retrospectively studied the qualitative tumor characteristics on preoperative MRIs. T1-weighted, T2-weighted, fluid-attenuated inversion recovery, and contrast-enhanced gradient-echo T1-weighted imaging were acquired after administering 0.1 ml/kg of gadolinium-based contrast material (gadodiamide, Omniscan; GE Healthcare AS, Oslo, Norway).

Two neurosurgeons (Y-KX and F-DY, with 15 and 10 years of experience, respectively) independently reviewed MR images for qualitative tumor characteristics. A senior neurosurgeon made all final decisions when the other two surgeons disagreed (WX-T, with 30 years of experience). The reviewers recorded the presence or absence of the following conventional MRI characteristics in a binary fashion: 1) cortical involvement, 2) multicentric tumor distribution, 3) uniformity, and 4) margin contrast enhancement. Cortical involvement was defined as any expansile T2 signal abnormality or contrast enhancement in the cerebral cortex. Multicentric tumor distribution was defined as involvement of multiple sites, not connected by obvious routes of spread. Uniformity was defined as more homogeneous or rough texture proportion if less than 33.3% on MRI T2-flair images. Margin contrast enhancement was categorized as being well-defined if perimeter of more than 66.7% on enhanced T1-weighted imaging.

### Survival Analyses

The interval from the date of initial diagnosis (data of first surgery) to the date of death was defined as overall survival (OS). The interval between the date of initial diagnosis (data of first surgery) to the progression of disease was defined as progression-free survival (PFS1). The interval from the date of first progression to death was defined as the overall survival of sGBM (OS2). The interval was censored at the last follow-up visit. The Response Assessment in Neuro-Oncology (RANO) criteria were used to define glioma progression, which incorporated both clinical status/deterioration and radiologic changes on MRI (2). The patients’ vital status and date of last follow-up were last updated on February 2, 2020.

Survival analyses (OS and OS2) were done using the Kaplan-Meier method. Survival distributions were compared using log-rank tests.

### Statistical Methods

Patients in the nomogram cohort were randomly divided into two groups (three patients in the derivation group and 1 in the validation group). Chi-square or t-tests were used to evaluate the differences between the two groups. Logistic regression analyses (univariate and multivariate) were used to identify patient risk factors. First, we performed univariate logistic regression analyses using all available clinical and biomarkers variables. Then, multivariate logistic regression analyses were performed using variables with *p*<0.05 from univariate analyses. Independent factors were selected from multivariate analyses and used to establish the nomogram.

All statistical analyses were performed with R version 3.5.1. The following packages were used: “survival” (Terry M Therneau), “plyr” (Hadley Wickham), “rms” (Frank E Harrell), “survminer” (Alboukadel Kassambara), “neuralnet” (Stefan Fritsch), “pROC” (Xavier Robin), “DynNom” (Amirhossein Jalali), “pHeatmap” (Raivo Kolde), and “stargazer” (Hlavac, 2018) (Marek Hlavac). In two-sided tests, P<0.05 was considered statistically significant.

### Nomogram Construct

A nomogram was formulated based on the results of the multivariate logistic regression analysis of the derivation group. To evaluate the nomogram performance, the area under (AUC) the receiver operating characteristic curve (ROC) curve was used to evaluate the sensitivity and specificity of the nomogram. A calibration curve was drawn as an indicator of internal calibration. An AUC value greater than 0.7 indicated good discrimination. Nomogram performance was further evaluated in the validation group *via* ROC and calibration curve.

## Results

### Patient Summary of Nomogram Cohort 

In the nomogram cohort, a total of 89 patients (55 males and 34 females) were included with a mean age of 38.55 years (range: 8–70 years old). A histopathological review of tumor specimens at initial diagnosis revealed 63 (70.8%) WHO grade II and 26 (29.2%) WHO grade III gliomas. Astrocytic molecular diagnosis (54, 60.7%) was more prevalent, while the remaining 35 tumors (39.3%) were oligodendroglial, and 40 of the 89 tumors were upgraded to sGBM after recurrence. In total, 25 of 89 tumors (28.1%) retained their former grade upon recurring while 64 (71.9%) progressed to a higher grade. Gross total resection was achieved in 74 cases (83.1%) and subtotal resection in 15 cases (16.9%). All patients received post-operative chemotherapy [temozolomide or PCV (procarbazine, lomustine, vincristine)] and radiotherapy (RT). The mean PFS after the first surgery was 1860 days (range: 177–6310 days). The mean follow-up interval was 3880 days (range: 744–6330 days).

**Figure 1:**
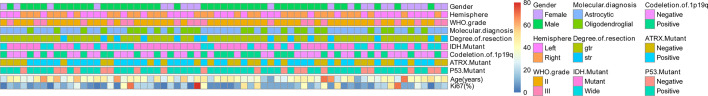
Nomogram cohort patient summary.

Patients were randomly divided into two groups (derivation group: 67 cases; validation group: 22 cases). The general characteristics of patients are given in **Table 1** and **Figure 1**. Patient characteristics were similar between the derivation and validation groups.

**Table 1:** Patient characteristics in the nomogram cohort.

		Derivation group	Validation group	P value
Number of patients, n (%)		67 (75.3%)	22 (24.7%)	
Gender, n (%)				0.07
	Male	45 (67.2%)	10 (45.5%)	
	Female	22 (32.8%)	12 (54.5%)	
Age in years, mean (range)		39.93 (9–70)	37.18 (8–60)	
Hemisphere, n (%)				0.29
	Left	34 (50.7%)	14 (63.6%)	
	Right	33 (49.3%)	8 (36.4%)	
Location, n (%)				
	Frontal lobe	18 (26.9%)	10 (45.5%)	0.10
	Temporal lobe	16 (23.9%)	4 (18.2%)	
	Parietal lobe	7 (10.4%)	2 (9.1%)	
	Insula lobe	4 (6.0%)	0	
	Thalamus	3 (4.5%)	0	
	Others	3 (4.5%)	1 (4.5%)	
	Multi-lobe	16 (23.9%)	5 (22.7%)	
WHO grade, n (%)				0.82
	II	47 (70.1%)	16 (72.7%)	
	III	20 (29.9%)	6 (27.3%)	
Molecular diagnosis at primary surgery, n (%)				**0.02**
	Astrocytic	42 (37.3%)	12 (22.7%)	
	Oligodendroglial	25 (28.4%)	10 (31.8%)	
Degree of resection, n (%)				0.33
	GTR	53 (79.1%)	21 (95.5%)	
	STR	14 (20.9%)	1 (4.5%)	
Progression-free survival in days, mean (range)		1746.22 (177–6310)	1975.00 (243–5982)	

GTR, gross total resection; STR, subtotal resection. The bold value highlights the significant values (P < 0.05).

### Univariate and Multivariate Analyses

According to a literature review and previous results from our research team, we focused on six clinical factors, six radiological factors, and four biomarkers as potential prognostic factors. Then, logistic regression analyses were performed in the derivation group to identify the utility of these potential prognostic factors. A univariate logistic regression analysis was used to explore the association between tumor transformation and each clinical radiological factor and biomarker. Among all six factors, two (molecular diagnosis and WHO grade) were significantly favorable clinical factors. Five of the six assessed radiological factors (location, cortical involvement, multicentric, uniformity, and margin enhance) demonstrated detectable differences. Among the biomarkers tested, only *1p19q* codeletion was found to be statistically significant. Synergy between three of the biomarkers (*IDH*+/*ATRX*-/*TP53*-) was also found to be statistically significant.

Multivariate logistic regression analyses of the clinical factors and candidate biomarkers were performed. Using this approach, WHO grade, molecular diagnosis, location, cortical involvement, multicentric, uniformity, margin enhance, *1p19q* codeletion, and biomarker synergy (*IDH*+/*ATRX*-/*TP53*-) were independent prediction factors (**Table 2**).

**Table 2:** Univariate and multivariate logistic regression analysis.

		Univariate analysis		Multivariate analysis	
		Odds ratio (95%CI)	p value	Odds ratio (95%CI)	p value
Clinical factors					
	Age		0.298		
	Gender		0.792		
	WHO grade (III vs. II)	5.294 (1.636–17.127)	**0.004**	4.461 (1.801–24.845)	**0.042**
	Molecular diagnosis (Astrocytic vs. Oligodendroglial)	2.832 (1.002–8.000)	**0.018**	16.780 (2.193–128.413)	**0.007**
	Degree of resection		0.164		
	PFS		0.563		
Radiological factors					
	Hemisphere		0.389		
	Location (Nonfrontal vs. frontal)	3.413 (1.227–9.434)	**0.017**	8.529 (1.916–37.977)	**0.002**
	Cortical involve (Negative vs. positive)	3.185 (1.170–8.696)	**0.028**	2.369 (1.059–7.407)	**0.036**
	Multicentric (Positive vs. Negative)	5.587 (1.391–22.437)	**0.015**	5.765 (2.819–17.315)	**0.047**
	Uniformity (Negative vs. positive)	2.864 (1.059–7.743)	**0.043**	3.937 (1.783–13.987)	**0.049**
	Margin enhance (Positive vs. Negative)	6.214 (2.022–19.098)	**0.001**	4.196 (1.156–15.042)	**0.026**
Biomarkers					
	IDH		0.433		
	Ki-67		0.101		
	1p19q Co-deletion (Negative vs. positive)	6.060 (2.045–17.857)	**0.001**	5.268 (1.108–25.056)	**0.003**
	ATRX		0.853		
	TP53		0.095		
	Synergy of IDH+/ATRX-/TP53- (Negative vs. positive)	14.286 (1.712–125.000)	**0.005**	12.022 (2.645–23.235)	**0.046**

The bold values highlights the significant values (P < 0.05).

Based on these findings, we established a sGBM prediction model for the derivation group, which was visualized as a nomogram.

### Nomogram Derivation, Validation, and Accessibility

**Figure 2:**
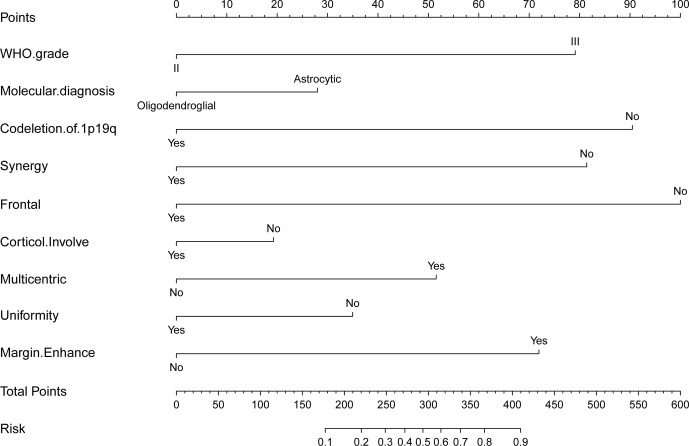
Nomogram for possibility of upgrade to sGBM at the following recurrence of lower grade gliomas.

**Figure 3:**
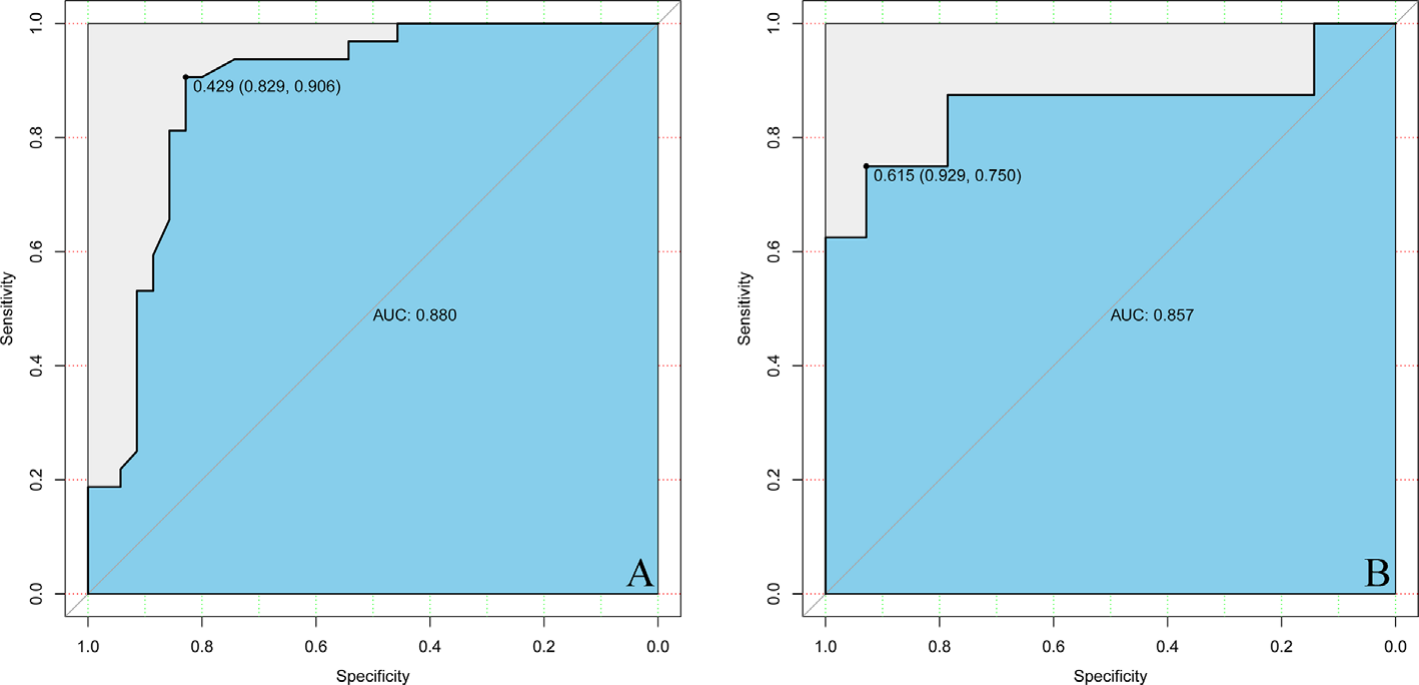
ROC curves of nomogram in the derivation **(A)** and validation **(B)** groups.

**Figure 4:**
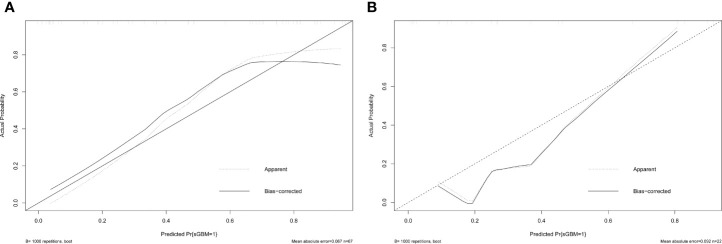
Calibration of nomogram in the derivation **(A)** and validation **(B)** groups.

Estimated possibilities for upgrading to sGBM were obtained according to the sum of each variable score (**Figure 2**). The sum of each variable point was plotted on the total points axis, and an estimated upgrade possibility was obtained by drawing a vertical line from the total points axis straight down to the outcome axis. A ROC curve was used to evaluate the sensitivity and specificity of the nomogram prediction model. The model had good sensitivity and specificity, with an AUC of 0.880 (**Figure 3A**). The calibration graph was drawn with the predicted the possibility of upgrade to sGBM (**Figure 4A**). This graph demonstrated good nomogram calibration.

To validate the nomogram’s stability, we also conducted a validation study using the validation group. The AUC of the nomogram in the validation group was 0.888, demonstrating a good prediction (**Figure 3B**). On a calibration graph, the validation group revealed a moderate calibration (**Figure 4B**).

For wider and easier use by clinicians and researchers, a dynamic program was generated from a normal nomogram and was uploaded to https://warrenwrl.shinyapps.io/DynNomapp/. By simply choosing the corresponding options of a clinical data of lower-grade glioma patients and pressing predict, the possibility of upgrading to sGBM in the next recurrence can be acquired.

### Clinical Utility of Nomogram on MGMT-Unmethylated Cohort

**Figure 5:**
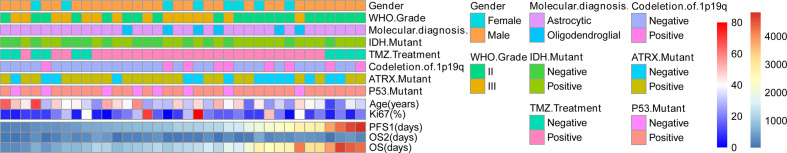
Survival analysis cohort patient summary.

A cohort of patient was summarized to validate the clinical utility of nomogram. Patients were divided into a high-risk group for those with an estimated possibility higher than 70% per the prediction model. According to the standard, 36 high-risk patients with unmethylated *MGMT* were enrolled in the cohort, all of whom were upgraded to sGBM upon tumor recurrence (**Figure 5**). These patients’ median age was 38.47 years (range 11–69). There were more male (28, 77.8%) than female subjects (8, 22.2%) in this cohort. A histopathological review of tumor specimens at initial diagnosis revealed 21 (58.3%) WHO grade II and 15 (41.7%) WHO grade III gliomas. Astrocytic gliomas (30, 83.3%) were the majority of these, while six (16.7%) tumors were oligodendroglial (**Table 3**). Overall, this cohort was representative of unmethylated *MGMT* and lower-grade gliomas, which had a higher risk of being upgraded to sGBM.

**Table 3:** Clinical characteristics of the unmethylated *MGMT* cohort.

Number of patients		36
Median age at diagnosis (range)		38.47 (11–69)
Male:Female ratio		3.5
Histologic grade (%)		
	*WHO II*	21 (58.3)
	*WHO III*	15 (41.7)
Molecular diagnosis (%)		
	*Astrocytic*	30 (83.3)
	*Oligodendroglial*	6 (16.7)
Adjuvant treatment after initial diagnosis		
	*TMZ+RT*	26 (72.2)
	*RT only*	10 (27.8)

**Figure 6:**
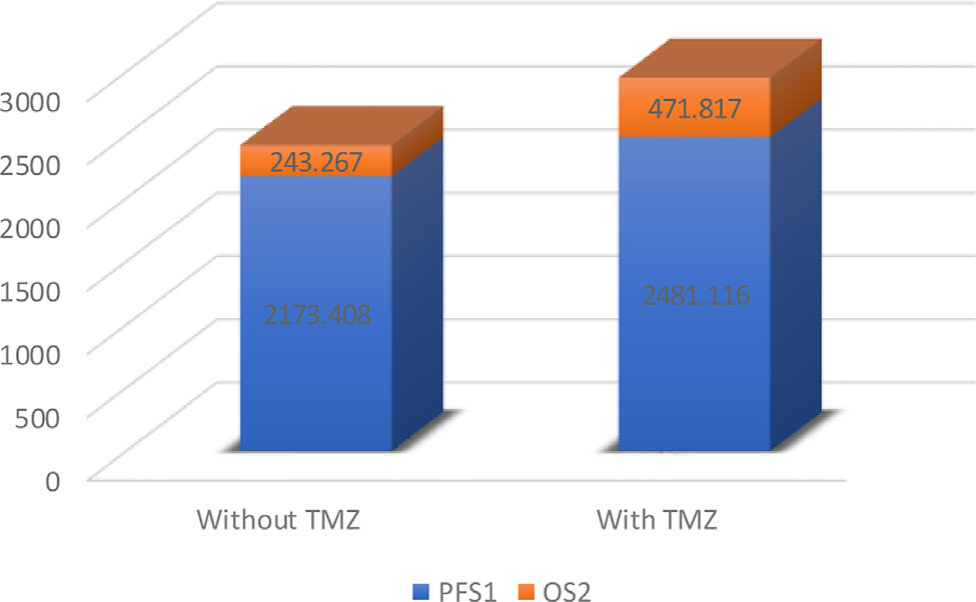
Mean overall survival of unmethylated *MGMT* glioma patients.

**Figure 7:**
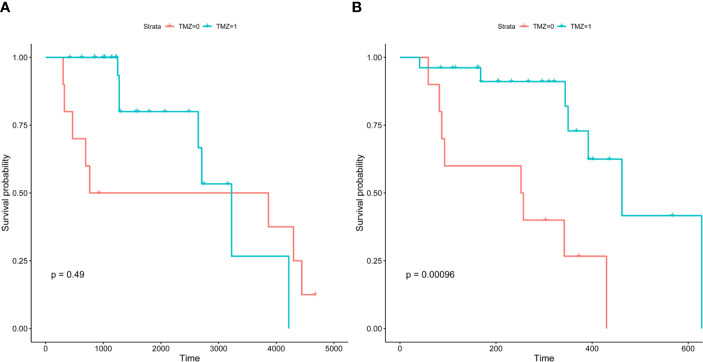
Kaplan-Meier survival curves of *MGMT-*unmethylated cohort. Precursor of sGBM possess a “lag response” to TMZ, which will reveal after the glioma recurrence.GBM, Glioblastoma; sGBM, Secondary glioblastoma; LGG, Lower grade glioma; WHO, World Health Organization; TMZ, Temozolomide; *MGMT*, O(6)-methylguanine-DNA methyltransferase; *IDH*, Isocitrate dehydrogenase; *ATRX*, alpha thalassemia/mental retardation syndrome X-linked.

Following their initial diagnosis, patients within the above cohort mostly underwent TMZ+RT treatment (26, 72.2%), while the remaining patients underwent RT as an adjuvant treatment (10, 27.8%). All patients underwent TMZ+RT treatment after their second diagnosis with sGBM. Consistent with the results of previous research, we observed a longer OS in those who underwent TMZ treatment compared to those who did not, though this was not significant (median OS 2952.933 days versus 2416.675 days, p=0.49, **Figure 6**, **Figure 7A**). However, when OS2 was compared separately (median OS 471.817 days versus 243.267 days), there was a significant difference in the unmethylated MGMT patients, who appeared to benefit from a former treatment with TMZ after tumor recurrence and upgrading to sGBM (p<0.001, **Table 4**, **Figure 7B**).

**Table 4:** Univariate and multivariate analysis using Cox Proportional Hazard model of variables influencing the overall survival and second overall survival.

		Univariate analysis	Multivariate analysis		
		p value	Odds ratio	95%CI	p value
OS	Gender	0.752			
	IDH (Wide vs. Mutant)	**0.042**	0.319	0.090–1.131	0.077
	1p19q Codeletion (Positive vs. negative)	0.678			
	ATRX	0.784			
	WHO grade	0.667			
	Adjuvant treatment (TMZ+RT vs. RT only)	0.491			
OS2	Gender	0.150			
	IDH (Wide vs. Mutant)	0.315			
	1p19q Co-deletion (Positive vs. negative)	**0.003**	13.781	2.205–86.124	**0.005**
	ATRX	0.825			
	WHO grade	0.145			
	Adjuvant treatment (TMZ+RT vs. RT only)	**0.001**	7.692	1.838–32.258	**0.005**

The bold values highlights the significant values (P < 0.05).

## Discussion

As a multicomponent process involving several genetic mutations, gliomagenesis has been abundantly researched (3). Longitudinal retrospective analyses of the processes underlying lower-grade glioma recurrence and progression have been instrumental in studying sGBM gliomagenesis.

### Key Molecular Markers for Defining Lower-Grade Glioma

As an early alteration in gliomagenesis, *IDH* presents in the majority of low‐grade glioma cases (4). A consensus on the distinct nature of *IDH* mutant gliomas and molecular heterogeneity among *IDH* wild-type gliomas has led to substantial revisions in the diagnostic categorization of adult diffuse gliomas. *1p19q* codeletion, where parts of oligodendroglial tumors exhibited a loss of heterozygosity in the short arm of chromosome 1 (*1p*) and the long arm of chromosome 19 (*19q*). *1p19q* codeletions in gliomas became a valuable biomarker of TMZ and PCV chemotherapy effectiveness and were associated with a better OS (5). For astrocytic tumors, which do not typically exhibit *1p19q* codeletions, mutations in the alpha thalassemia/mental retardation (*ATRX*) gene could help to maintain telomere length (6, 7). *TP53* is a key DNA damage repair molecule whose molecular mutation is associated with astrocytic histology and thus increases microglia infiltration (8).

Given the synthesis of these key alterations, we created this behavior-oriented classification system for lower-grade gliomas. This system was designed to stratify lower grade gliomas based on the different outcomes at their recurrence and established a basis for further longitudinal analyses of tumor upgrading pattern. In further studies, the online prediction program may powered by artificial intelligence for further automatically fusion multicenter data and acquiring self-learning and correcting ability to better indicate clinical individual treatment.

### Behavior-Oriented Predictions May Alter Treatment Paradigms

*MGMT* encodes O^6^-methylguanine-DNA methyltransferase, an enzyme that repairs the DNA damage inflicted by alkylating agents such as TMZ and nitrosourea-based chemotherapies (9). Decades of cross-sectional OS-oriented research have revealed that *MGMT* promoter methylation indicates a better response to TMZ therapy. This has led to a consensus in North America and Europe that TMZ could be excluded as a standard treatment for unmethylated *MGMT* glioma patients (10). However, as the present study indicates, the OS of sGBM is better by using TMZ in previous lower-grade tumors, even in unmethylated *MGMT* glioma patients. The precursor of sGBM may possess a “lag response” to TMZ, which the TMZ was employed on the original treatment stage (OS, **Figure 7A**), but the response will reveal after the glioma recurrence (OS2, **Figure 7B**). Despite the fact that the underlying mechanism remains unclear, TMZ-based interventions may alter sGBM gliomagenesis and result in OS differentiation. Due to discrepancies between cancer staging classifications and glioma grading systems, longitudinal behavior-oriented research on lower-grade glioma stratification is critical and insufficient.

Moreover, early-stage glioblastomas may develop into bulky mass lesions within a few weeks. Patients with such lesions often have a dismal prognosis (11). Clinicians may therefore consider individualized follow-up plans for each patient. For higher-risk individuals upgrading to sGBM evaluated by the nomogram, early and frequent MRI re-examinations could be recommended and may improve sGBM prognosis by discovery and intervention in the early stages of the disease progression.

### Is Succumbing to sGBM a Settled Destiny? 

Cancer risk factors have previously been found, such as cigarette smoking, alcohol use, and excess body weight. However, few carcinogenic risk factors for glioma have been identified. Distinct pathways have been identified as core drivers of gliomagenesis (12–14). *IDH* and *ATRX* mutations, *1p19q* codeletions, and *TP53* mutations are early events in gliomagenesis, and the tumor behavior of lower-grade gliomas can be predicted depending on these key molecules.

A question in the literature is whether upgrading to sGBM is inevitable in some cases. Two potential answers to this question were examined. First, before lower-grade gliomas form, alternations of key mutations have already occurred. Since the upgrade to sGBM is affected only by these key alternations, the destiny of succumbing to sGBM is embedded in the process of gliomagenesis. Second, we assessed whether there is a fateful point at which lower-grade gliomas undergo a malignancy transformation to sGBM. Besides key alternations, some interventions such as surgery or chemotherapeutics may influence tumor fate. For latter theories, we may have chances to hold back the malignant transformation, and have a glimpse of preventing gliomagenesis.

### Limitations

Despite its significant implications, the present study had several limitations. First, long periodic longitudinal follow-up should be implemented for a larger sample size. For our future work, we aim to expand our cohort to eventually be able to precisely depict the genesis paradigm from lower grade glioma to sGBM. Second, due to funding and tissue limitations we were limited in the number of biomarkers detected. Future studies should be done to expand the biomarker detection range to identify additional potential mutations.

## Conclusions 

Clinical and radiological factors, as well as biomarker-based and behaviorally-oriented nomograms, may offer a feasible stratification paradigm for lower grade gliomas. Use of such an approach will assists neurosurgeons in sorting lower grade glioma patients for improved individualized treatment plans.

## Data Availability Statement

The datasets generated for this study are available on request to the corresponding author.

## Ethics Statement

The studies involving human participants were reviewed and approved by The First Affiliated Hospital of Zhengzhou University and West China Hospital of Sichuan University. Written informed consent to participate in this study was provided by participants.

## Author Contributions

X-TW was responsible for the research design. R-LW and J-GL participated in the clinical data collection. F-DY and Y-KX participated in MRI evaluation. R-LW and L-WZ participated in the sample collection, experiment performing and statistical analysis. R-LW write the manuscript. R-LW contributed to manuscript submission. All authors have contributed to manuscript. All authors contributed to the article and approved the submitted version.

## Conflict of Interest

The authors declare that the research was conducted in the absence of any commercial or financial relationships that could be construed as a potential conflict of interest.
